# Association between gastroesophageal reflux and bruxism: a scoping and bibliometric review

**DOI:** 10.1590/1807-3107bor-2026.vol40.046

**Published:** 2026-07-27

**Authors:** Vitor Laguardia Guido FARIA, Luiz Renato PARANHOS, Douglas Teixeira da SILVA, Danilo Cassiano FERRAZ, Paulo Isaias SERAIDARIAN, Marcelo Bighetti TONIOLLO, Rafael Rodrigues LIMA, Paulo Cézar SIMAMOTO JÚNIOR

**Affiliations:** (a) Universidade Federal de Uberlândia – UFU, Graduate Program in Dentistry, Uberlândia, MG, Brazil; (b) Universidade Federal de Uberlândia – UFU, School of Dentistry, Department of Orthodontics, Uberlândia, MG, Brazil.; (c) University of Pittsburgh, School of Dental Medicine, Department of Endodontics, Pittsburgh, PA, USA.; (d) Pontifícia Universidade Católica de Minas Gerais – PUC-MG, Department of Dentistry, Belo Horizonte, MG, Brazil.; (e) Universidade de Rio Verde – UniRV, Dental School of Rio Verde, Rio Verde, GO, Brazil.; (f) Universidade Federal do Pará – UFPA, Institute of Biological Sciences, Laboratory of Functional and Structural Biology, Belém, PA, Brazil.; (g) Universidade Federal de Uberlândia – UFU, School of Dentistry, Department of Prosthesis and Dental Materials, Uberlândia, MG, Brazil.

**Keywords:** Bruxism, Gastroesophageal Reflux

## Abstract

This review summarizes the literature on association between gastroesophageal reflux disease (GERD) and bruxism. A systematic search was performed in June 2024 and updated in October 2025 across electronic databases (MedLine/PubMed, LILACS/BBO, SciELO, EMBASE, Scopus, and Web of Science) and grey literature (OpenGrey, ProQuest, and LIVIVO). Case reports, clinical trials, and observational studies addressing the GERD-bruxism association in individuals of any age without systemic diseases, syndromes, or neuromotor disorders were included. Study selection and data extraction were independently conducted by two reviewers. Twenty-two articles met inclusion: six case reports, six cross-sectional studies, four case-control studies, and six clinical trials, totaling 15,889 participants (73.4% women; mean age 44.5 years). All but three cross-sectional studies reported an association between GERD and bruxism. Clinical studies have consistently demonstrated that esophageal acidification, whether experimentally induced or present in patients with GERD, increases the hourly frequency of rhythmic masticatory muscle activity (RMMA), short bursts and RMMA with grinding. Two clinical trials that tested proton pump inhibitors observed a reduced RMMA frequency. One clinical study assessed awake bruxism, and two observational studies assessed sleep bruxism. Bibliometric analysis identified 9 author clusters and 17 collaboration networks, with research concentrated in Japan but showing marked global growth from 2018 to 2025. Bruxism and GERD showed a positive association. However, methodological heterogeneity and numerous confounders prevented robust evidence and limited the clinical inference. Bibliometric analysis shows that research remains centered in Japan but is expanding globally, reflecting growing interdisciplinary interest and the need for greater international and methodological integration.

## Introduction

Gastroesophageal reflux disease (GERD) and bruxism have considerable global prevalence, varying from 2.5 to 51.2% and around 10 to 20% in Europe and the USA.^1^ Sleep bruxism (SB) prevalence ranges across 9.7 to 15.9%,^2^ while possible awake bruxism (AB) varies between 16 to 32 %,^3^ based on the employed diagnostic method.^4^

The latest international bruxism consensus redefined the terms SB and AB. SB is masticatory muscle activity during sleep, characterized by rhythmic (phasic) or nonrhythmic (tonic) and is not a movement disorder or a sleep disorder. AB is a “masticatory muscle activity during wakefulness, characterized by repetitive or sustained tooth contact and/or by bracing or thrusting of the mandible and is not a movement disorder.^5^ Bruxism and GERD may share common factors such as sleep quality, obstructive sleep apnea, and psychological disorders such as stress, anxiety, and depression.^6,7^Furthermore, bruxism has been hypothesized to exert a protective effect by activation mechanism of the salivary glands through masticatory muscle movements.^8^ Thereby, acts as a resource to neutralize intraoral pH during gastroesophageal reflux events. However, the hypothesis remains uncertain.^9^

Although GERD is not an exclusive sleep disorder, it is more frequent and intense during sleep.^6^ Thus, it has been associated with sleep fragmentation, mainly due to conscious awakening caused by reflux symptoms or amnestic arousal. One study reported that reflux events occurred most often immediately after microarousal. The authors hypothesized that the lower esophageal sphincter transiently relaxes after arousal, which might explain the increase in acid reflux events.^10^ SB presents a complex pathophysiology modulated by serial events before rhythmic masticatory muscle activity (RMMA), such as autonomic cardiac activation (four to eight minutes before RMMA), increased electroencephalographic activity (four seconds before RMMA), tachycardia (one second before RMMA), altered respiratory activity, and microarousals.^6^ Arousals from sleep may represent a permissive window for the onset of RMMA episodes.^6^

A systematic review with a meta-analysis of observational studies confirmed a strong association between reflux and SB and between SB and GERD/reflux.^11^ However, observational studies have examined this association by administering proton pump inhibitors (PPIs) in GERD patients^12^ and the association between GERD and AB and SB through experimental acidification of the gastroesophageal tract in healthy individuals.^13,14^ These studies could be integrated and analyzed together with those included in the aforementioned systematic review.^11^ Thereby, providing additional insights into the association between GERD and bruxism.

To fill this gap, this study aimed to explore the potential association between GERD and bruxism to improve the diagnosis and treatment based on all types of evidence available in the literature. It has two specific objectives: 1) to conduct a scoping review to identify and analyze existing literature, and 2) to verify these findings through bibliometric analysis assessing characteristics and trends in published scientific research on this topic.

## Methodology

### Protocol and registration

This scoping review was based on the Joanna Briggs Institute (JBI) manual recommendations^15^ and reported according to the Preferred Reporting Items for Systematic Reviews and Meta-Analyses extension for Scoping Reviews (PRISMA-ScR).^16^ The review protocol was registered in the Open Science Framework database (https://doi.10.17605/OSF.IO/E2VYD).

The bibliometric analysis followed BIBLIO checklist for reporting bibliometric reviews of the biomedical literature.^17^

### Eligibility criteria

This scoping review was based on the following question: What is known about the association between gastroesophageal reflux/acidification and bruxism in adults? The questions followed the PCC acronym: Population – children, adolescents, and adult patients with SB or AB; Concept - the association of gastroesophageal reflux/acidification with bruxism; and Context - any healthcare or community setting.

The inclusion criteria were studies reporting animal, laboratory, case report, case series, clinical, and observational studies with human individuals of any age, and investigations establishing the association between acidification of the gastroesophageal tract and SB and AB. This study considered both objective (upper digestive endoscopy, polysomnography, pH monitoring, actigraphy, EMG devices for bruxism, and dental wear indices) and subjective (survey) methods. There were no restrictions on the language or publication year. Exclusion criteria were studies involving participants with other systemic diseases, syndromes, or neuromotor problems, or pregnant women.

### Search and information sources

Electronic searches were conducted up to June 2024 and updated in October 2025 in MedLine (via PubMed), Scopus, Web of Science, EMBASE, LILACS/BBO, and SciELO databases. OpenGrey, ProQuest (for theses and dissertations), and LIVIVO platforms partially captured the grey literature. The references of the eligible articles were carefully analyzed. This search model aimed to minimize selection bias. The MeSH (Medical Subject Headings), Embase Subject Headings (Emtree), and DeCS (Health Science Descriptors) provided search descriptors. Boolean operators “AND” and “OR” were combined keywords, respecting the syntax rules of each database (Table 1).

**Table 1:** Strategies for database searches.

Database	Search strategy June 2024 - (updated in October 2025)
**Main Databases**	
MedLine (via PubMed)	**#1** “sleep bruxism”[Mesh Term] OR “bruxism” [Mesh Term] OR “nocturnal bruxism”[tw] OR “sleep-related bruxism”[tw] OR “awake bruxism”[tw] OR “bruxer*”[tw] OR “tooth grinding”[tw] OR “tooth clenching”[tw] OR “teeth grinding”[tw] OR “teeth clenching”[tw] OR “teeth grinding disorder[tw] OR “jaw clenching”[tw]
http://www.ncbi.nlm.nih.gov/pubmed/	**#**2 “gastroesophageal reflux”[Mesh Term] OR “gastro oesophageal reflux”[tw] OR “GERD”[tw] OR “GORD”[tw] OR “oesophageal reflux”[tw] OR “gastro-oesophageal reflux disease”[tw] OR “acid reflux”[tw] OR “esophageal acid perfusion”[tw] OR “gastric acid reflux”[tw] OR “gastric acid reflux disease”[tw] OR “stomach ulcer”[tw] OR “gastric ulcer”[tw] OR “stomach damage”[tw] OR “stomach lesion”[tw] OR “erosive esophagitis”[tw] OR “reflux esophagitis”[tw]
	**#1 AND #2**
Scopus	**#1** TITLE-ABS-KEY (“sleep bruxism” OR “bruxism” OR “nocturnal bruxism” OR “sleep-related bruxism” OR “awake bruxism” OR “bruxer” OR “bruxers” OR “tooth grinding” OR “tooth clenching” OR “teeth grinding” OR “teeth clenching” OR “jaw clenching” OR “mandibular parafunction”)
http://www.scopus.com/	**#2** TITLE-ABS-KEY (gastroesophageal reflux disease” OR “gastro oesophageal reflux” OR “GERD” OR “GORD” OR “oesophageal reflux” OR “gastro-oesophageal reflux disease” OR “acid reflux” OR “esophageal acid perfusion” OR “gastric acid reflux” OR “gastric acid reflux disease” OR “stomach ulcer” OR “gastric ulcer” OR “stomach damage” OR “stomach lesion” OR “erosive esophagitis” OR “reflux esophagitis”)
	**#1 AND #2**
Web of Science	**#1**TS =(sleep bruxism) OR TS =(bruxism) OR TS =(nocturnal bruxism) OR TS =(sleep-related bruxism) OR TS =(bruxer) OR TS =(bruxers) OR TS =(tooth grinding) OR TS =(tooth clenching) OR TS =(teeth grinding) OR TS =(teeth clenching) OR TS =(jaw clenching) OR TS =(mandibular parafunction)
http://apps.webofknowledge.com/	**#2**TS =(gastroesophageal reflux disease) OR TS =(Gastro oesophageal Reflux) OR TS =(GERD) OR TS =(GORD) OR OR TS =(oesophageal reflux) OR TS =(gastro-oesophageal reflux disease) OR TS =(acid reflux) OR TS =(esophageal acid perfusion) OR TS =(gastric acid reflux) OR TS =(gastric acid reflux disease) OR TS =(stomach ulcer) OR TS =(gastric ulcer) OR TS =(stomach damage) OR TS =(stomach lesion) OR TS =(erosive esophagitis) OR TS =(reflux esophagitis)
	**#1 AND #2**
Embase	**#1** (‘sleep bruxism’/exp OR ‘sleep bruxism’ OR ‘bruxism’/exp OR ‘bruxism’ OR ‘nocturnal bruxism’/exp OR ‘nocturnal bruxism’ OR ‘sleep-related bruxism’/exp OR ‘sleep-related bruxism’ OR ‘awake bruxism’/exp OR ‘awake bruxism’ OR ‘bruxer’ OR ‘bruxers’ OR ‘tooth grinding’/exp OR ‘tooth grinding’ OR ‘tooth clenching’/exp OR ‘tooth clenching’ OR ‘teeth grinding’/exp OR ‘teeth grinding’ OR ‘teeth clenching’/exp OR ‘teeth clenching’ OR ‘jaw clenching’ OR ‘mandibular parafunction’)
http://www.embase.com/	**#2** (‘gastroesophageal reflux disease’ OR ‘gastro oesophageal reflux’ OR ‘gerd’ OR ‘gord’ OR ‘oesophageal reflux’ OR ‘gastro-oesophageal reflux disease’ OR ‘acid reflux’ OR ‘esophageal acid perfusion’ OR ‘gastric acid reflux’ OR ‘gastric acid reflux disease’ OR ‘stomach ulcer’ OR ‘gastric ulcer’ OR ‘stomach damage’ OR ‘stomach lesion’ OR ‘erosive esophagitis’ OR ‘reflux esophagitis’)
	**#1 AND #2**
LILACS / BBO	**((**“sleep bruxism” OR “bruxism” OR “nocturnal bruxism” OR “sleep-related bruxism” OR “awake bruxism OR “bruxer” OR “bruxers” OR “tooth grinding” OR “tooth clenching” OR “teeth grinding” OR “teeth clenching” OR “jaw clenching” OR “mandibular parafunction”) AND (“gastroesophageal reflux disease” OR “gastro oesophageal Reflux” OR “GERD” OR “GORD” OR “oesophageal reflux” OR “gastro-oesophageal reflux disease” OR “acid reflux” OR “esophageal acid perfusion” OR “gastric acid reflux” OR “gastric acid reflux disease” OR “stomach ulcer” OR “gastric ulcer” OR “stomach damage” OR “stomach lesion” OR “erosive esophagitis” OR “reflux esophagitis”))
http://lilacs.bvsalud.org/	

### Data charting process

The findings from the primary databases were exported to EndNote Web™ software (Clarivate™ Analytics, Philadelphia, USA). The program automatically excluded duplicates and the remaining duplicates were manually removed. Other results were exported to Rayyan QCRI (Qatar Computing Research Institute, Doha, Qatar)^18^ to select and evaluate titles and abstracts. Grey literature studies were analyzed manually, first removing duplicates and then reading the titles and abstracts on Microsoft Word™ 2010 (Microsoft™ Ltd., Washington, USA). Two reviewers firstly discussed the inclusion and exclusion criteria, subsequently performed a calibration exercise by assessing 20% of articles to determine inter-rater agreement. Selection was initiated after reaching an adequate level of agreement (Kappa ≥ 0.81).

In the first stage of the review, two examiners (V.L.G.F. and D.T.S.) independently evaluated titles according to the eligibility criteria. Titles that were unrelated to the review proposal were discarded. The reviewers evaluated the abstracts and read those that were considered eligible. A third reviewer (L.R.P.) helped to evaluate and resolve disagreements between the reviewers. Potentially eligible studies were retrieved and analyzed. Furthermore, the reference lists of all the retrieved studies were read to search for eligible studies.

### Data collection

The reviewers performed a calibration exercise by analyzing 20% of the retrieved articles to ensure consistency, coherence, and agreement during data extraction prior to data collection. Two reviewers (V.L.G.F. and D.T.S.) independently extracted the data from the eligible studies, and a third reviewer (L.R.P.) analyzed the conflicts.

General data extracted from the articles included study identification (author, year, country, and study type) and sample characteristics (number, distribution by sex, average age, body mass index (BMI), alcoholism, and sleep and psychological disorders). Specific information included diagnostic methodologies for GERD and bruxism, experimental and gastroesophageal pH assessment methods, and primary and secondary study outcomes.

Studies that provided participants’ weight and height but did not report BMI were calculated as stipulated by the World Health Organization (WHO) by dividing body weight (kilograms) by height (square meters).^19^

In cases of unavailable data, the authors were contacted via e-mail. Regarding missing or incomplete data and the absence of further clarification from the study authors, a ‘no information’ annotation was applied.

### Data synthesis and analysis

The included articles were divided into two categories to standardize the outcome comparisons: a) observational studies and b) experimental clinical studies. Bruxism was analyzed and divided into two domains: sleep and awake bruxism. Microsoft Excel™ 2019 (Microsoft™ Ltd., Washington, USA) spreadsheets organized the data collected from the eligible studies. Descriptive and qualitative analyses were used to report the study characteristics, frequency of outcomes, and assessment methods for bruxism and gastroesophageal reflux. The summarized mean and 95% confidence interval were calculated for the average age of the sample participants.

### Bibliometric analysis

The eligible studies were imported into EndNote X9 and exported as an RIS file for analysis in VOSviewer 1.6.15 (Centre for Science and Technology Studies, Leiden University, Netherlands) and Power BI (Microsoft, Redmond, WA, USA). Each article provided the following data: publication year, authors, country, journal, and impact factor, supplied by the Journal Citation Reports (JCR, Clarivate Analytics, https://jcr.clarivate.com/jcr/). Co-authorship connections were grouped into clusters and country data were based on author affiliations to assess international collaborations. GraphPad Prism 9.5.1 (Boston, MA, USA) was used to visualize the journals and countries with the most publications on this topic.

## Results

### Evidence source selection

**Figure 1:**
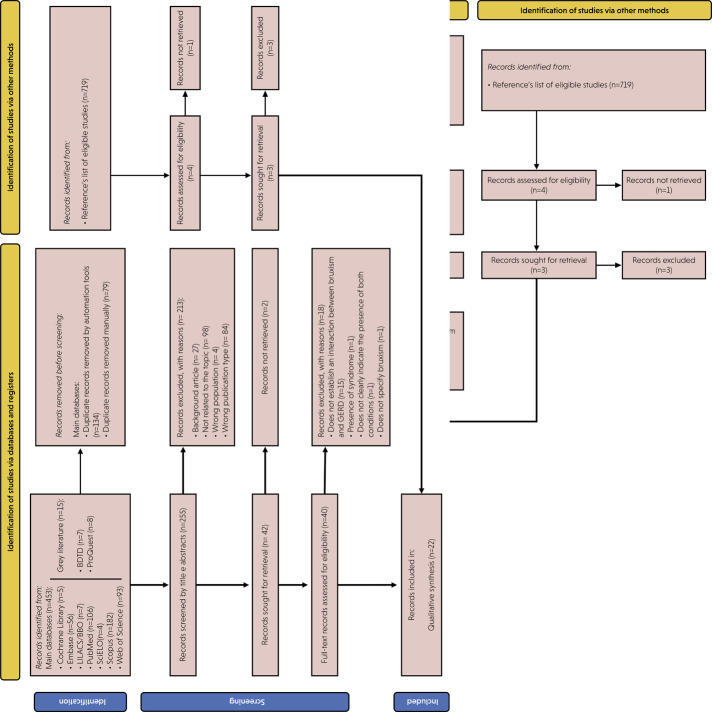
Flowchart of the selection process according to PRISMA.

The first phase of study selection identified 468 records distributed across nine electronic databases, including grey literature. After removal of duplicates, 255 results remained for title and abstract screening. After review, 40 studies were selected for a full-text reading. Subsequently, unrelated articles not as per the inclusion criteria were excluded, resulting in a final inclusion of 22 studies. Figure 1 illustrates the search, identification, inclusion, and exclusion of eligible studies. Table 2 lists the studies excluded after full-text readings along with the reasons for exclusion.

**Table 2:** Studies excluded after full-text readings and reasons for exclusion (n = 18).

	Excluded study	Reason for exclusion
1	*Smith & Rafeek, 2008*	Does not establish an association between bruxism and GERD
2	*Khois., 2009*	Presence of syndrome (OSA)
3	*Sakaguchi et al., 2014*	Does not establish an association between bruxism and GERD
4	*Scaramucci et al., 2014*	Does not establish an association between bruxism and GERD
5	*Noble et al., 2015*	Does not clearly indicate the presence of both conditions
6	*Arman et al., 2016*	Isolated prevalence of each condition
7	*Peşkersoy et al. - 2016*	Does not establish an association between bruxism and GERD
8	*Bernardes et al., 2019*	Does not establish an association between bruxism and GERD
9	*Li et al., 2019*	Does not specify bruxism
10	*Zuza et al., 2019*	Does not establish an association between bruxism and GERD
11	*Blumer et al., 2022*	Does not establish an association between bruxism and GERD
12	*Iqbal et al. 2022*	Does not establish an association between bruxism and GERD
13	*McEloy, 2023*	Does not establish an association between bruxism and GERD
14	*Mendieta-Hernández et al., 2023*	Does not establish an association between bruxism and GERD
15	*Khan., 2023*	Does not establish an association between bruxism and GERD
16	*Ramos, 2023*	Does not establish an association between bruxism and GERD
17	*Jiménez-Núñez et al., 2024*	Does not establish an association between bruxism and GERD
18	*Sun et al., 2024*	Does not establish an association between bruxism and GERD

### Characteristics of the included studies

**Figure 2:**
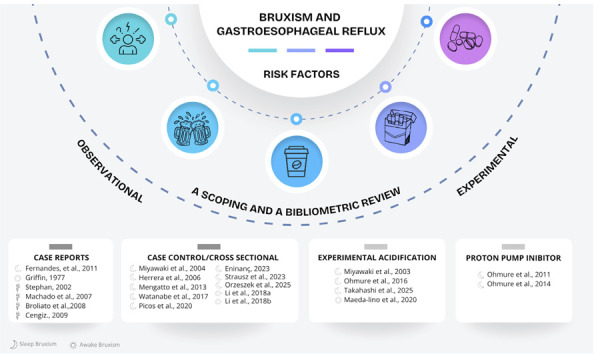
Diagram of the association between bruxism and GERD and their related risk factors.

The publication intervals of the included studies ranged from 1977 to 2025. Most selected articles were conducted in Japan,^12-14,20-24^ followed by China,^6,25^ Brazil,^7,26-29^ Turkey,^30,31^ USA,^32,33^ Finland^34^ and Poland^35^ and one multicenter study was performed in two countries: France and Romania.^36^ The included studies assessed 15.889 participants, with 26.6% (4.217) men and 73.4% (15.889) women. The participant ages varied with a mean of 44.5 years. A total of six case reports^26-29,31,32^, six cross-sectional^7,22,33-36^ studies, four case-control^6,20,25,30^ studies, and six clinical^12-14,21,23,24^ studies were included. SB was assessed in one case report^29^, four case-control^6,20,25,30^ studies, six cross-sectional^7,22,33-36^ studies, and four clinical studies.^12-14,21^ AB was evaluated independently in one case report^32^ and two clinical^23,24^ studies and simultaneously with SB in two case-control studies.^6,25^ The type of bruxism was not specified in four case reports^26-28,31^. Only one case report^32^ and one observational^33^ study analyzed the association between SB and GERD in children without syndromes or other health conditions. Few studies have reported participants’ BMI, with a mean of 21.94, indicating normal weight for the sample, according to the WHO.^19^ Alcoholism,^6,22,25,30,35^ sleep disorders,^6,34^ and psychological disorders,^6,7,32,34^ such as anxiety, depression and stress, were the most frequent risk factors. Figure 2 provides a schematic illustration of the association between bruxism and GERD, along with their associated risk factors. Studies used different methods to evaluate bruxism, such as electromyography (EMG) of masticatory muscles^12-14,20,21,23,24^ audio and video recordings,^12-14,20,21^ polysomnography (PSG),^12-14,33,35^ questionnaires,^7,23-25,30,36^ and clinical evaluations.^6,7,22,25-33,36^ Esophagogastroduodenoscopy,^12,26^ endoscopy,^7,12,22,27,28^ and trans nasal probe with pH sensors on a portable endoscope,^13,14,20,21,23,33^ investigated acidification of the gastroesophageal tract, Other studies have reported the use of clinical assessments,^22,29,31,32,35^ and questionnaires,^6,7,12,22,25,30,35,36^ to diagnose GERD (Table 3).

**Table 3:** Main characteristics of the eligible studies.

Author	Country						Risk factors	Assessment method for GERD or gastroesophageal acidification	Assessment method for bruxism
		Study type	Sample/ type of bruxism investigated	Sex	Age (mean/SD or MD)	BMI (mean)	Alcohol, Caffein comsuption, Smokinga; Sleep disordersb; Psychological disordersc		
Griffin, 1977	USA	Case Report	1	1 ♂	7	*	Emotional stress	History of regurgitation associated with emotional stress and abdominal pain.	History and clinical evaluation
			Awake bruxism						
Stephan, 2002	Brazil	Case Report	1	1 ♂	29	*	Normal acid drinks or food stuffs consumption. No drugs or alcohol use.	Esophagogastroduodenoscopy,	History and clinical evaluation
			Bruxism*						
Miyawaki et al., 2003	Japan	Case-control	G1 (cases - with bruxism) = 10	10 ♂	G1 = 27 ± 7.0	20.9	*	Transnasal probe with pH sensor	EMG and audio-video recordings
			G2 (controls - without bruxism) = 10	10 ♀	G2 = 26.4 ± 4.7				
			Sleep bruxism						
Miyawaki et al., 2004	Japan	Prospective clinical study	Without bruxism = 8	8 ♂	24 ± 2.1	*	*	Transnasal probe with pH sensor	EMG and audio-video recordings
			With bruxism = 4	4 ♀					
			Sleep bruxism						
Herrera et al., 2006	USA	Cross-sectional	20	10 ♂	5 – 15 (9.2 ± 3.2)	*	*	pH-probe testing	PSG; Clinical evaluation
			Without bruxism = 10	10 ♀					
			With bruxism = 10						
			Sleep bruxism						
Machado et al., 2007	Brazil	Case Report	1	1♀	42	*	Smoker	Endoscopy	Clinical evaluation
			Bruxism*						
Broliato et al., 2008	Brazil	Case Report	1	1 ♂	22	*	*	Endoscopy	Clinical evaluation
			Bruxism*						
Cengiz et al., 2009	Turkey	Case Report	1	1 ♂	54	*	*	Clinical history	Clinical evaluation
			Bruxism*						
Fernandes et al., 2011	Brazil	Case Report	1 Sleep bruxism	1 ♂/	37/	*	*	Medical evaluation/	Clinical evaluation
			1 Sleep bruxism	1 ♀	18			Endoscopy	Clinical evaluation
Ohmure et al., 2011	Japan	Cross-over	G1 (experimental night/saline infusion) = 6	12 ♂	24.2 ± 2.8	21.4	*	Transnasal probe with pH sensor	PSG, EMG and audio-video recordings
			G2 (saline infusion/experimental night) = 6						
			Sleep bruxism						
Mengatto et al., 2013	Brazil	Cross-sectional	G1 (GERD) = 19	13 ♂	44.6 ± 14.0	25.1	c: Stress: 51.1% of the sample	Questionnaire and endoscopy	Questionnaire and clinical evaluation
			G2 (Non-GERD) = 26	32♀			(63.2% GERD group; 42.3% non-Gerd group)		
			Sleep bruxism						
Ohmure et al., 2014	Japan	Quasi-experimental	Healthy adult men	15 ♂	23.6 ± 4.3	21.9	*	Transnasal probe with pH sensor	PSG, EMG and audio-video recordings
			Sleep bruxism						
Ohmure et al., 2016	Japan	Cross-over	G1 (placebo/PPI) = 6	5 ♂	30.3 ± 7.9	*	*	Endoscopy, Esophagogastroduodenoscopy, Questionaire	PSG, EMG and audio-video recordings
			G2 (PPI/placebo) = 6	7 ♀					
			Sleep bruxism						
Watababe et al..2017	Japan	Retrospective Cross-sectional	G1 (GERD) = 105	90 ♂	G1: 66.4 ± 13.0	G1: 22.9 ± 3.6	a (alcohol): G1: 27.6%	Clinical evaluation and	Clinical history
			G2 (Non-GERD) = 50	65 ♀	G2: Older 63.8 ± 8.2	G2: Older 22.8 ± 4.3	G2: Older 24%	gastrointestinal fiberscope	
			Sleep bruxism		Younger: 28.7 ± 2.6	Younger: 22.1 ± 3.3	Younger: 12%		
Li et al., 2018a	China	Case-control	G1 (cases - with bruxism) = 887	887 ♂	G1 = 27 (22-37)	G1 = 21.1	a(alcohol): 17.3%	Questionnaire	Questionnaire and clinical evaluation
			G2 (control – without bruxism) = 887	887 ♀	G2 = 28 (22-37)	G2 = 20.8	n = 308 (G1 = 158; G2 = 150)		
			Sleep and awake bruxism				b: 39.5% (48.7% G1; 39.4% G2)		
							n = 742 (423 G1; 310 G2)		
							c: Depression:		
							% = 14.3% (18.6% G1; 10% G2)		
							n = 254 (165 G1; 89 G2)		
							Anxiety:		
							% = 8.1% (11.2% G1; 5.2% G2) n = 145 (99 G1; 46 G2)		
Li et al., 2018b	China	Case-Control	G1 (cases - with bruxism) = 363	294 ♂	G1 = 28 (22 - 38)	G1 = 21.4	20.6% (21.5% G1; 19.8% G2)	Questionnaire	Clinical evaluation
			G2 (control – without bruxism) = 363	432 ♀	G2 = 29 (23 - 39)	G2 = 21.0	n = 150 (78 G1; 72 G2)		
			Sleep and awake bruxism						
Maeda-lino et al., 2020	Japan	Cross-over	12 healthy men	12 ♂	24.1 ± 4.6	21.9	*	Transnasal probe with pH sensor	EMG, Questionnaire
			Awake bruxism						
Picos et al., 2020	France and Romania	Cross-sectional	G1 – GERD	100 ♂	43 (median age)	*	*	Questionnaire	Questionnaire and clinical evaluation
			patients =141	163 ♀					
			G2 - non-GERD						
			controls =122						
			Sleep bruxism						
Eninanç, 2023	Turkey	Case-control	G1 (cases - with bruxism) = 200	200 ♂	G1 = 27.17 ± 8.060	*	a(alcohol): G1 = 21 (10.5%)	Questionnaire	Questionnaire and clinical evaluation
			G2 (control – without bruxism) = 200	200 ♀	G2 = 25.94 ± 6.945		G2 = 14(7%)		
			Sleep bruxism						
Strausz et al., 2023	Finland	Cross-sectional	12.297 (Bruxism)	2427 ♂	51 ± 15.8	*	b(insomnia): 2.7%	International Classification of Diseases	International Classification of Diseases
			1370 (GERD and Bruxism)	9821 ♀			c(anxiety): 10.6%		
			Sleep Bruxism				c(depression): 22.3%		
		
Orzeszek et al., 2025	Poland	Cross-sectional	114 TMD patients	72 ♂	21–71 (37.67)	*	a(alcohol): 36.84%	History	PSG
			Sleep bruxism	42 ♀			a(caffein): 22.8%		
							a(smoking): 10.53%		
Takahashi et al., 2025	Japan	Quasi-experimental	10 healthy men	8 ♂	18 – 49 (25.4 ± 2.8)	*	*	Transnasal probe with pH sensor	EMG, Questionnaire
			Awake bruxism	2 ♀					

♂: male; ♀: female; *Data not provided by the authors. G1 – group 1; G2 – group 2; EMG: Electromyography; PSG: Polysomnography; TMD: Temporomandibular disorders.

### Study types

#### a) Observational studies

The remaining articles showed an association between bruxism and GERD, considering the cross-sectional and case-control studies, with the exception of Herrera et al^33^, Picos et al.^36^, and Orzeszek et al.^35^ The analytical methods varied among studies, as odds ratio calculations were performed in the studies by Mengatto et al.^7^, Li et al.^6,25^, and Strausz et al..^34^ In the studies by Watanabe et al.^22^, Picos et al.,^36^ and EniNanç^30^ bivariate association tests were conducted to compare prevalence rates. However, the association was not quantified. In a study by Orzeszek et al.,^35^ the authors did not find significant differences in the frequency or intensity of bruxism in relation to GERD based on group comparisons. Miyawaki et al.^21^ did not apply a direct association test between SB and GERD. Instead, they performed within-group comparisons (paired t-tests or Wilcoxon tests) to examine the differences in physiological measures related to bruxism and reflux episodes. Through an analysis of the frequency of RMMA, short bursts, and teeth-clenching episodes, they reported that these were significantly higher during periods of esophageal pH drop than at other times. In contrast, Herrera et al.,^33^ through the analysis of RMMA alone, found no relationship between SB and GERD in a pediatric sample through the analysis of RMMA alone. SB was the most frequently investigated type, as AB was evaluated alongside SB only in the studies by Li et al..^6,25^ The case-control study by Li et al.^6^ documented a statistically significant association between GERD and all types of bruxism, isolated (SB and AB) and combined (SB + AB), after adjusting for confounding variables (p < 0.001). Li et al.^25^ also identified a statistically significant association between the two conditions. However, no differentiation between the bruxism types was reported, providing only an overall odds ratio (OR) (Table 4).

**Table 4:** Main findings of the observational studies.

Author, year	Type of bruxism	GERD and bruxism	GERD	Bruxism	Dental tightening frequency (times/h)	Short-burst EMG frequency (times/h)	RMMA frequency (times/h)	Relationship GERD and Bruxism	Outcome
Miyawaki et al., 2004	Sleep	*	*	n = 4	Without pH drop = 0.1 ± 0.1	Without pH drop = 2.0 ± 2.2	Without pH drop = 1.3 ± 1.0	Frequency RMMA; short-burst, and clenching episodes	Positive association (p <0.01)
					With pH drop = 0.7 ± 1.1	With pH drop = 8.6 ± 2.9	With pH drop = 12.4 ± 7	On pH decrease: 12.4(7); 8.6(2.9); 0.7(1.1)	
								Other times: 1.3(1); 2.0(2.2); 0.1(0.1)	
Herrera et al., 2006	Sleep	n = 2	*	n = 10	*	*	6 ± 5	Pearson correlation	Negative correlation (p > 0.05)
Mengatto et al., 2013	Sleep	n = 14	n = 5	n = 6	*	*	*	SB: OR = 6.58; 95% CI [1.40-30.98]	Positive association (p = 0.017)
Watanabe et al., 2017	Sleep	n = 18	n = 87	n = 2	*	*	*	Prevalence SB	Positive association (p = 0.041)
								Non-GERD: 23.1%	
								GERD: 73.7%	
Li et al., 201^8^a	Sleep and awake	n = 155	n = 23	n = 732	*	*	*	SB: OR = 6.71, 95% CI (4.22–10.68)	Positive association (p < 0.001)
								AB: OR 13.06, 95% CI [5.32–32.05]	
								SB+AB: OR = 6.48, 95% CI [3.05–13.77]	
Li et al., 2018^b^	Sleep and awake	n = 68	*	n = 295	*	*	*	OR = 5.30, 95% CI [2.62-10.70]	Positive association (p < 0.001)
Picos et al., 2020	Sleep	n = 83	*	n = 50	*	*	*	Prevalence SB	Negative association (p = 0.166)
								GERD: 58.8%	
								Control: 41.2%	
Eninanç., 2023	Sleep	n = 29	n = 5	n = 171	*	*	*	BS and GERD: 14.5%	Positive association (p < 0.000)
								BS Non-GERD: 85.5%	
								GERD and Non-Bruxism: (2.5%)	
								Non-GERD and Non-Bruxism (97.5%)	
Strausz et al., 2023	Sleep	1.370	26.184	12.297	*	*	*	OR = 2.06 95% CI [1.94-2.19]	Positive association (p < 1×10^-4^)
Orzeszek et al., 2025	Sleep	33	*	90	*	*	*	Prevalence SB	Negative association (p = 0.542)
								GERD: 28,95%	

*information not reported; RMMA: rhythmic masticatory muscle activity; EMG: electromyography; GERD: gastroesophageal reflux disease. Odds Ratio.

The cross-sectional study by Strausz et al.^34^ evaluated the association between SB and GERD in a large population-based sample (n = 377,277) drawn from the FinnGen and UK Biobank cohorts, using health records coded according to the International Classification of Diseases (ICD). Phenotypic and genetic analyses were performed, including logistic regression and LD Score Regression adjusted for age, sex, and behavioral factors. The results demonstrated significant phenotypic and genetic correlations between SB and GERD, with no evidence of a direct causal relationship or a specific genetic overlap between the conditions.

Except for Griffin Jr,^32^ all the other case reports have primarily focused on the oral rehabilitation of patients with advanced tooth wear associated with bruxism and GERD.

#### b) Experimental studies

Among the experimental studies, crossover clinical trial^12,13,20,23^ designs were observed, as well as quasi-experimental models.^14,24^ Two studies^12,20^ tested PPIs, and four^13,14,23,24^ tested acid solution infusions in the gastroesophageal tract (0.1 N HCI, pH 1.2). Ohmure et al.^12^was the only study on SB using the esophageal acidification methodology and identified a significant increase in masseter muscle activity during acid infusion compared to the control group. Many other studies that also evaluated SB,^12,20^ tested the effect of PPIs and documented statistically significant reductions in RMMA frequency. None of the studies simultaneously evaluated both types of bruxism. Two studies^23,24^ evaluated AB using the esophageal acidification methodology and documented statistically significant increases in RMMA and masseter muscle activity after acid infusion compared with saline infusion. In the study by Takahashi et al.,^24^ the authors employed a faster infusion rate and a larger volume (10 mL/min for 10 minutes; total of 100 mL) to observe more pronounced effects and their temporal changes. Masseter muscle activity increased significantly compared with pre-infusion levels and decreased 10 and 20 min after acid infusion. This study did not provide mean and standard deviation values; instead, the results were presented graphically as medians without specific interquartile ranges for each time point. Ohmure et al.^13^ and Ohmure et al.^12^measured the EMG burst frequency. The distinct experimental methodology demonstrated a significant reduction in EMG bursts on the second night after PPI administration and an increase during acid infusion (Table 5).

**Table 5:** Main findings of the clinical trials.

Author, year	Bruxism type	RMMA frequency (times/h)		RMMA frequency with grinding (times/h)	Short-burst EMG frequency	RMMA frequency (times/h)		RMMA frequency with grinding (times/h)		Short-burst EMG frequency		Outcome
PROTON PUMP INHIBITOR												
Miyawaki et al., 2003	Sleep	Bruxism:	Control:	*		Bruxism:	Control:	*		*		Positive association (p < 0.01)
		pH< 3 = 0.0 ± 0.0 pH<4 = 0.0 ± 0.0 pH<5 = 0.1 ± 0.3	pH< 3 = 0.1 ± 0.3 pH<4 = 0.5 ± 0.9 pH<5 = 3.6 ± 1.6			-Placebo: 6.0 ± 2.2	-Placebo: 1.9 ± 3.2					
						-PPI: 3.7 ± 1.9	-PPI: 1.0 ± 0.6					
Ohmure et al., 2016	Sleep	*		*	*	**Night 1**	**Night 2**	**Night 1**	**Night 2**	**Night 1:**	**Night 2:**	
						-Placebo: 6.3 ± 3.1	-Placebo: 6.1 ± 3.3	-Placebo: 1.6 ± 1.6	Placebo: 3.2 ± 2.9	-Placebo: 59.4 ± 30.5	-Placebo: 59.7 ± 33.1	Positive association RMMA freq. 2nd night (p = 0.012)
						-PPI: 4.8 ± 3.8	-PPI: 4.8 ± 2.3	-PPI: 1.3 ± 2.2	-PPI: 2.2 ± 2.6	-PPI: 45.7 ± 36.5	-PPI: 46.0 ± 21.7	Frequency of EMG bursts 2nd night (p = 0.025)
Ohmure et al., 2011	Sleep	Baseline		**Baseline**	**Baseline**	**S.I.**	**A.I.**	**S.I.**	**A.I.**	**S.I.**	**A.I.**	Positive association Frequency of RMMA (p = 0.008)
		0.1 ± 0.2		14.6 ± 11.7	1.4 ± 1.1	1.3 ± 1.2	4.7 ± 4.2	0.2 ± 0.4	1.2 ± 1.1	12.1 ± 9.2	49.0 ± 33.0	Frequency of EMG bursts (p = 0.002) Positive correlation (p=0.020)
				**Total masseter muscle activity (%.s)**		**Total masseter muscle activity (%.s)**						
				**Baseline**		**S.I.**	**A.I.**					
Ohmure et al., 2014	Sleep			2668 ± 1619		2778 ± 173	3370 ± 2148					
Maeda-Lino et al., 2020	Awake			Reading task: 4105 ± 2852		Reading task: 4187 ± 2601	Reading task: 5180 ± 3558					Positive association (p = 0.019)
				Calculation task: 4887 ± 2407		Calculation task: 5033 ± 1784	Calculation task: 6053 ± 2744					Reading: (p = 0.583) Calculating: (p = 0.875)
Takahashi et al., 2025	Awake			*		*	*					Positive association (p < 0.001)

*Data not available; RMMA: rhythmic masticatory muscle activity; EMG: electromyography; A.I.: acid infusion; S.I: saline infusion.

In the study by Miyawaki al.^20^ the authors used the term “association”. However, they performed statistical comparisons using t-tests and Mann–Whitney tests. No specific tests of association between variables were conducted, indicating that the use of the term “association” was descriptive rather than statistically justified. In contrast, Ohmure et al.^13^ performed a correlation analysis between masticatory muscle activity and other variables using Spearman’s correlation test. The remaining studies did not explicitly describe the relationship between bruxism and GERD.

All the studies that conducted experimental acidification of the gastroesophageal tract^13,14,23,24^ included healthy individuals. Regarding blinding processes, four studies^13,14,23,24^ blinded the participants, two^12,20^ reported double blinding, and one^23^ did not perform blinding. The follow-up time varied between studies, as three^14,23,24^ occurred in a single day, one^20^ in two nights (with a one-week interval in between), one^13^ in four nights, and one^12^ in six nights. Table 4 presents the primary outcomes of experimental clinical studies.

## Bibliometric analysis

**Figure 3:**
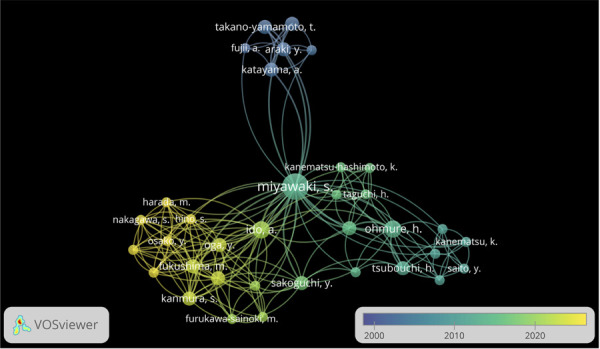
Co-authorship networks in the eligible studies. The image shows the grouping of five clusters forming the most solid network. Labels of different colors represent year of publication and line thickness the link-strength between co-authors.

Authorship analysis identified nine clusters with 108 authors distributed across 17 clusters. The five clusters demonstrated strong internal linking (Figure 3). The most connected authors within this group were S. Miyawaki (seven articles included, nine links) and A. Ido (n = 3,27 links).

**Figure 4:**
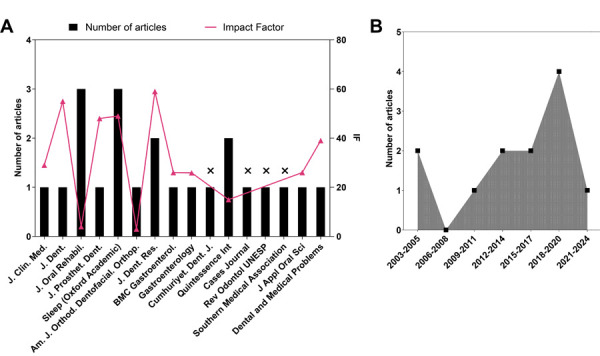
Graphical representation of the distribution of eligible articles by (a) journals, according to the prevalence of published articles and their corresponding impact factor in the Journal Citation Reports. Journals marked with × do not have an available impact factor in the Journal Citation Reports; and (b) publication trends over time.

Figure 4 summarizes the number of articles published on this topic categorized by journal, impact factor, and publication trend over time. *The Journal of Oral Rehabilitation* and *Sleep* published the most articles (n = 3 each) (Figure 4A). All journals had an impact factor of 5–25.9. Publication trends over time demonstrated that most eligible studies were published over the past ten years, with 2021-2025 being the most productive period for articles on GERD and bruxism (n = 5), followed by 2018-2020 (n = 4).

**Figure 5:**
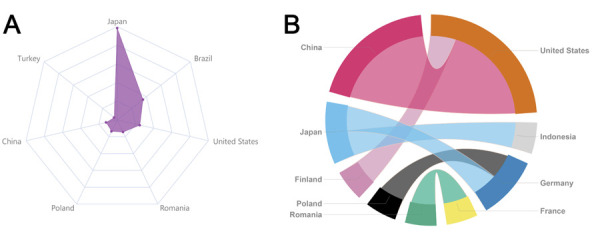
(a) Radar chart illustrating the prevalence of articles across countries with the eligible authors. Data points near the center represent lower article or citation counts, while those closer to the outer edge indicate higher counts. (b) The chord diagram visualizes the international collaboration among eligible studies, with thicker lines indicating higher citation weights between collaborations.

Regarding the geographic distribution, Japan led with 8 publications on esophageal acidification and bruxism, followed by Brazil and the United States, with five and three publications, respectively (Figure 5A). The largest international collaboration network was between China and the United States (n = 2) (Figure 5B).

## Discussion

Almost all observational studies showed an association between GERD and bruxism, except for three cross-sectional studies.^33,35,36^ All clinical studies showed that esophageal acidification, either experimentally or in patients with GERD, increased the frequency (times/h) of RMMA, short bursts, and RMMA with grinding.

Several studies included in this scoping review reported an association between GERD and both forms of bruxism. However, investigations specifically addressing AB remain scarce. The pathophysiology differs between SB and AB: psychosocial factors such as stress and anxiety play a greater role in AB, while SB is more frequently associated with alcohol and caffeine consumption, smoking, psychotropic medications, and GERD itself.^30^ The hypothesis is that both conditions share common risk factors and neurobiological mechanisms within the brain–gut axis that influence visceral sensitivity, gastrointestinal motility, and permeability.^37^ Cytokines resulting from chronic esophageal inflammation in GERD may also affect the central nervous system, contributing to psychological disorders^37^. In turn, stress-related biomarkers such as cortisol, as well as self-reported measures, support a positive association between stress, bruxism, and GERD.^38^ Li et al.^6^ found that depression, anxiety, and poor sleep quality partially mediated this association, with stronger relationship in women, possibly due to estrogen-related^39^ relaxation of the lower esophageal sphincter and increased reflux susceptibility.

Although smoking and alcohol use are more prevalent among men^30^, most studies did not statistically control for sex, and three studies included only male participants^13,14,23^. For example, Maeda-Iino et al.,^23^ For example, Maeda-Iino et al. used an all-male sample to avoid confounding effects related to hormonal and genetic variability. Lifestyle factors such as alcohol and caffeine consumption and smoking were often examined as exogenous risks. However, only Eninanç^30^ reported significantly higher smoking and caffeine use among bruxism patients, whereas Li et al.^6^ and Watanabe et al.^22^ found no significant associations. These discrepancies may result from low alcohol intake among study participants, cross-sectional designs, small sample sizes and limited statistical power.^22^ Ethanol, nicotine, and caffeine have been associated with bruxism and GERD^40-42^. These substances can disrupt sleep and mood, increase masticatory activity, and impair esophageal function, thereby promoting reflux symptoms.

BMI has also been correlated with exacerbated GERD symptoms due to greater intra-abdominal pressure and altered gastric positioning from visceral fat accumulation.^43^ However, the few studies in this review that evaluated BMI^7,22,25^ found no significant relationship with bruxism, likely because their samples consisted of individuals within the normal weight range.

Studies^33,35,36^ with an association between bruxism and GERD may be justified by the fact that the association was not the primary research objective. In addition, GERD was not confirmed using definitive diagnostic methods, and Herrera et al.^33^ employed a particularly small pediatric sample.

Previous literature supports the physiological association between the two conditions: exposure of the esophagus to gastric juice, composed of hydrochloric acid, pepsin, and enzymes active below pH 4^44^, reduces epithelial resistance^45^ and triggers salivation through the esophageal–salivary reflex via vagal stimulation, inducing reflex salivation.^46^ In GERD and SB, arousal and swallowing episodes increase RMMA, enhancing oral lubrication, mucosal protection, and acid neutralization^47^. The clinical studies included in this scoping review that tested experimental acidification of the gastroesophageal tract reported a significant increase in the occurrence of bruxism. Maeda-Iino et al.^23^ observed that intraesophageal acid infusion heightened masseter and sympathetic activity while suppressing parasympathetic responses, whereas psychological stress alone did not affect muscle activity. These findings suggest that patients with GERD may present greater daytime masticatory activity and a higher predisposition to AB.

It is important to note that the acid infusions (0.1 N HCl, pH 1.2) used in experimental models do not fully replicate the complexity of gastric juice, which contains multiple chemical components.^44^ Furthermore, these experiments were performed in healthy individuals, which limits their applicability to symptomatic GERD populations.^14,23,24^ Two clinical studies^12,20^ demonstrated that PPI therapy significantly reduced the frequency of RMMA compared with placebo, indicating that acid suppression not only relieved GERD symptoms and promoted mucosal healing, but also improved sleep quality by decreasing arousal frequency and prolonging the REM stage.^48^

Despite the predominance of SB research, only five studies^12-14,33,35^ employed PSG for diagnosis. PSG objectively records masticatory muscle activity during sleep through EMG, allows differentiation of RMMA from other orofacial or sleep-related movements.^49^ Additionally, it correlates these muscle events with sleep stages and arousal patterns, ensuring diagnostic accuracy^49^. Its limited use likely reflects accessibility issues, the need for specialized training, and high operational costs^50^. Most experimental studies and one observational study^21^ utilized EMG, which quantifies the RMMA, short bursts, and grinding frequency. However, diagnosing AB remains challenging because EMG recordings in awake individuals may not fully capture masticatory activity patterns^51^.

Bruxism and GERD are the potential risk factors for tooth wear^2^. Clinical history and examinations are the most common diagnostic tools for both disorders. Dental wear analysis has been frequently investigated, particularly in studies centered on tooth surface loss, where data on bruxism and GERD were collected given their shared outcomes. Most case reports have described patients presenting with both conditions requiring extensive oral rehabilitation due to generalized tooth wear, highlighting the clinical significance of their association.

Bibliometric analysis identified Japan as the epicenter of research and collaboration on the association between GERD and bruxism, with highly influential contributors such as Miyawaki and Ido leading cohesive author networks that have produced extensive experimental evidence on esophageal acidification and masticatory activity. Asian researchers appear particularly engaged in this topic, likely influenced by environmental and epidemiological factors that increased basal and stimulated gastric acid secretion in Japan between the 1970s and the 1990s,^52^ along with declining *Helicobacter pylori* infection rates that have heightened GERD susceptibility.^53^ Since 2018, there has been a marked global increase in publications, reflecting growing interdisciplinary interest across dentistry, sleep medicine, and gastroenterology, as evidenced by the diversity of high-impact journals addressing this topic. Despite Japan’s leading role, the asymmetric geographic distribution highlights emerging, yet limited, intercontinental collaborations, suggesting opportunities for broader international engagement and greater scientific representativeness in future studies.

This review identified the limitations of the studies that should be addressed. A limited number of investigations, mostly observational, and only a few clinical trials were identified, and there was a further lack of information. Regarding the available clinical studies, only Ohmure et al.^12^ included patients diagnosed with GERD, while three^14,20,23^ experimentally induced esophageal acidification in healthy individuals, which did not accurately reflect real clinical conditions. These studies also showed substantial methodological variability, including heterogeneous variable categorization, inconsistent data collection and analysis procedures, unclear diagnostic criteria, lack of specification of the bruxism type investigated, and inconsistent reporting of findings. Importantly, many studies failed to analyze or report relevant risk factors and most relied on questionnaires or self-reports to diagnose bruxism and GERD, limiting the reliability and scientific validity of the associations observed.

Future research should prioritize randomized controlled trials, cohort studies, and double-blind crossover designs with longer follow-up periods, including patients with GERD confirmed by digestive endoscopy and evaluated by dentists using objective diagnostic tools such as polysomnography. Regardless of the study type, the shared risk factors of GERD and bruxism should be systematically assessed using appropriate statistical models to clarify their influence. Additionally, greater attention should be given to AB, which remains underexplored compared with SB. Accurate diagnosis can be improved through ecological monitoring, as smartphone-based applications have enabled the real-time assessment of masticatory muscle activity, while advances in data analysis continue to enhance reliability^51^. Self-reports, third-party observations, and clinical examinations (e.g., tooth wear and muscle hypertrophy) can support the diagnosis; however, these indicators lack specificity and may also result from other conditions, such as GERD triggering RMMA, based on our eligible studies.

### Clinical implications

Evidence suggesting a bidirectional relationship between GERD and bruxism underscores the importance of integrated diagnostic and therapeutic approaches. Dental professionals should consider screening for reflux symptoms in patients presenting with signs of bruxism, particularly in those with unexplained dental wear, muscle fatigue, or morning discomfort. Physicians managing GERD should be aware of the potential orofacial manifestations associated with this condition. Early identification and multidisciplinary management involving dental and medical teams may prevent the progression of mucosal and dental damage, improve sleep quality, and reduce symptom recurrence. Moreover, controlling acid exposure through pharmacological or behavioral measures, along with interventions aimed at stress reduction and sleep hygiene, may play a complementary role in mitigating bruxism episodes and enhancing overall patient outcomes.

## Conclusion

Bruxism and GERD are positively associated, as esophageal acidification experimentally induced or present in patients with GERD increases the frequency (times/h) of RMMA, short bursts, and RMMA with grinding. Moreover, PPI appeared to reduce the RMMA frequency. However, heterogeneity and varied confounders limited clinical inferences. Bibliometric analysis remained centered to Japan but is expanding globally, underscoring growing interdisciplinary interest and the need for greater international and methodological integration.

## Data Availability

The authors declare that all data generated or analyzed during this study are included in this published article.
